# PCA-based unsupervised feature extraction for gene expression analysis of COVID-19 patients

**DOI:** 10.1038/s41598-021-95698-w

**Published:** 2021-08-30

**Authors:** Kota Fujisawa, Mamoru Shimo, Y.-H. Taguchi, Shinya Ikematsu, Ryota Miyata

**Affiliations:** 1 School of Life Science and Technology, Tokyo Institute of Technology, Tokyo, 152-8550 Japan; 2 Graduate School of Engineering and Science, University of the Ryukyus, Okinawa, 903-0213 Japan; 3 Department of Physics, Chuo University, Tokyo, 112-8551 Japan; 4 Department of Bioresources Engineering, National Institute of Technology, OkinawaCollege, Okinawa, 905-2192 Japan; 5 Faculty of Engineering, University of the Ryukyus, Okinawa, 903-0213 Japan

**Keywords:** Computational science, Machine learning

## Abstract

Coronavirus disease 2019 (COVID-19) is raging worldwide. This potentially fatal infectious disease is caused by severe acute respiratory syndrome coronavirus 2 (SARS-CoV-2). However, the complete mechanism of COVID-19 is not well understood. Therefore, we analyzed gene expression profiles of COVID-19 patients to identify disease-related genes through an innovative machine learning method that enables a data-driven strategy for gene selection from a data set with a small number of samples and many candidates. Principal-component-analysis-based unsupervised feature extraction (PCAUFE) was applied to the RNA expression profiles of 16 COVID-19 patients and 18 healthy control subjects. The results identified 123 genes as critical for COVID-19 progression from 60,683 candidate probes, including immune-related genes. The 123 genes were enriched in binding sites for transcription factors NFKB1 and RELA, which are involved in various biological phenomena such as immune response and cell survival: the primary mediator of canonical nuclear factor-kappa B (NF-*κ*B) activity is the heterodimer RelA-p50. The genes were also enriched in histone modification H3K36me3, and they largely overlapped the target genes of NFKB1 and RELA. We found that the overlapping genes were downregulated in COVID-19 patients. These results suggest that canonical NF-*κ*B activity was suppressed by H3K36me3 in COVID-19 patient blood.

## Introduction

Coronavirus disease 2019 (COVID-19) is an infectious disease caused by severe acute respiratory syndrome coronavirus 2 (SARS-CoV-2). It was first identified in December 2019 in Wuhan, Hubei, China, and has resulted in an ongoing pandemic^1–3^. COVID-19 is a potential zoonotic disease with a moderate mortality rate (2–5%) and is primarily transmitted through droplets and direct contact with infected individuals or incubation carriers^4^. The large number of mild and asymptomatic cases is considered to be a feature of SARS-CoV-2^5–7^. However, it can severely impact the lungs, and COVID-19 survivors can suffer long-term health effects. Although numerous studies on COVID-19 have been conducted, our understanding of it is still far from complete. Currently there are no clearly effective preventive or therapeutic remedies for COVID-19. Patients with COVID-19 have no choice but to receive supportive care to relieve symptoms^8^. Therefore, it is imperative to elucidate the mechanism of COVID-19 and find an effective treatment method.

The “silver bullet” approach requires analyzing RNA-Seq data containing RNA extracted from samples. By comparing the gene expressions of COVID-19 patients with those of non-patients, we can obtain more information about the infectious disease pathology. A data-driven approach using machine learning is an efficient strategy for predicting mechanisms that are difficult to elucidate through the application of conventional knowledge-based analysis in biology. Although it is not difficult to obtain various kinds of omics data for COVID-19, the data is difficult to analyze because they often include several tens of thousands of candidate genes and few samples.

Recently, an unsupervised feature extraction method based on principal component analysis (PCA) has been suggested for its utility in gene selection. This method, called PCAUFE^9–27^, enables analysis of data sets with a small number of samples and many variables. The algorithm, which is based on linear algebra, is computationally light and has been confirmed to work well for various gene selection problems. For example, an integrated analysis of the mRNA/miRNA expression associated with posttraumatic-stress-disorder- (PTSD-) mediated heart disease^17^ and various cancers^12^ identified a possible candidate gene associated with those diseases. More recently, an integrated gene expression analysis of blood from patients with dengue hemorrhagic fever by using PCAUFE identified 46 genes that are critical to the disease progression, whereas other methods of bioinformatic analysis were unable to obtain such results^27^. Furthermore, a theoretical justification for the PCAUFE methodology was already developed in previous studies (for more details, see^27^).

**Figure 1:**
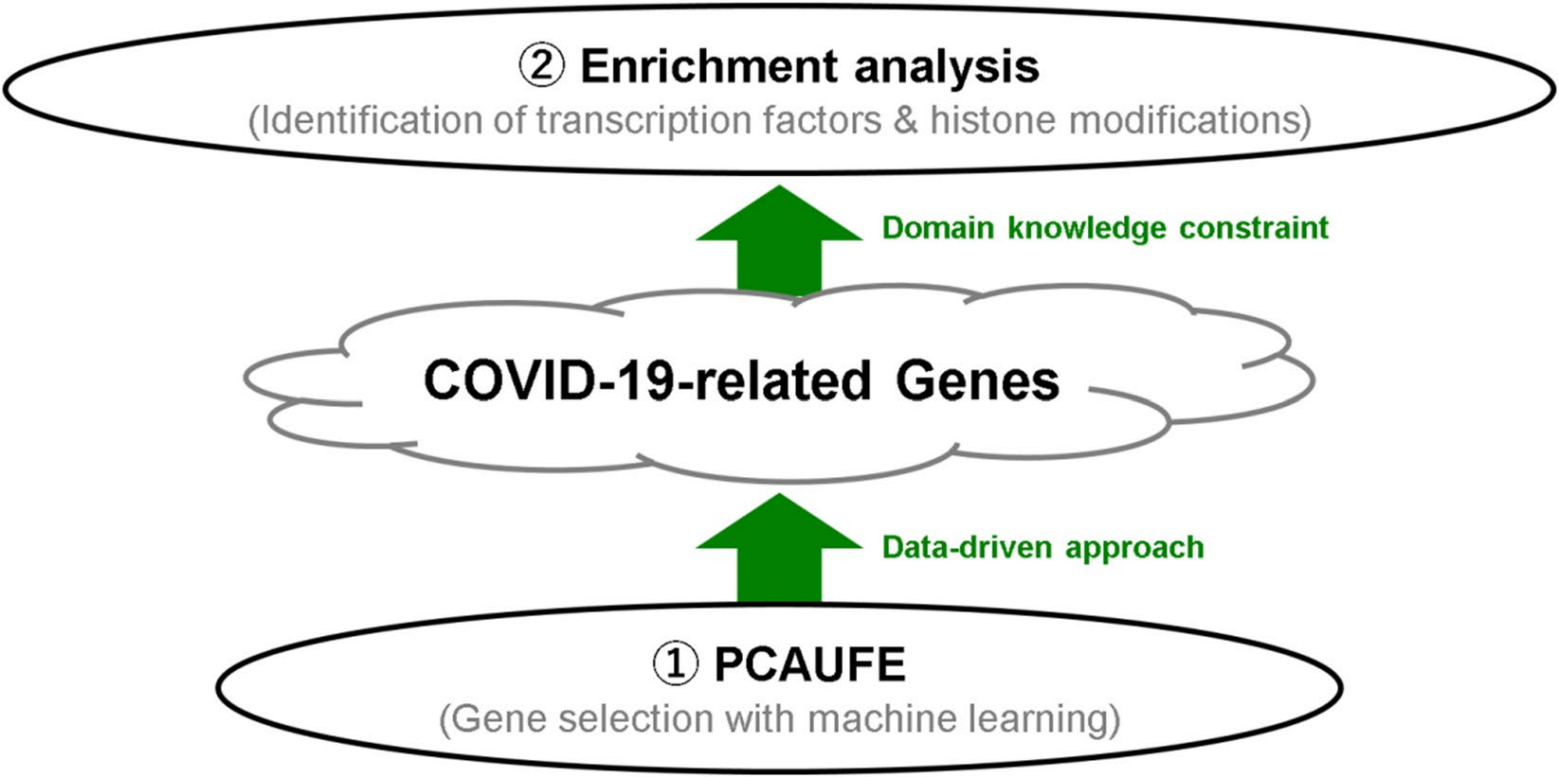
Outline of this study. First, we select genes related to the disease by using unsupervised machine learning; then, we enrich them by applying biological knowledge to identify the transcription factors and histone modifications of the selected genes.

In this paper, we identify genes associated with COVID-19 by applying PCAUFE to the RNA expression profiles of COVID-19 patients and healthy control subjects. Figure 1 shows an outline of our study. We confirm the reliability of the identified genes from the viewpoints of both biology and machine learning. Furthermore, we use bioinformatics tools to identify the transcription factors and histone modifications that regulate the selected genes in the upper layers. The novelty in this manuscript lies our findings that the application of PCAUFE to the gene expression profiles provided the smallest number of genes which are reasonable to explain the COVID-19 development from both machine learning and biological perspectives by comparing to the other typical gene selection methods described in the following section.

## Results

### Application of PCAUFE to gene expression in COVID-19 patients

This section describes how we used PCAUFE to analyze the gene expression patterns of multiple COVID-19 patients.

The first example (data set 1, GSE152418) was obtained by Arunachalam et al^28^. It includes five severity categories: ICU patients (IP), severe patients (SP), moderate patients (MP), convalescent patients (CP), and healthy controls (HC). By investigating the principal component (PC) loadings that statistically differentiated the group of IP + SP + MP (16 patients) from the group of CP + HC (18 non-patients), we found the second and third PCs (PC2 and PC3). The *P* values computed with a *t*-test rejected the null hypothesizes that the mean loadings within the group of IP+SP+MP and within the group of CP+HC were identical: 9*.*69 × 10^*−*5^ for PC2 and 3*.*67 × 10^*−*3^ for PC3. Although the PC1 loadings were the significantly different between patients and non-patients, the *P* value (1*.*83 × 10^*−*2^) was larger than those of PCs 2 and 3. As also shown in Fig. S1, the 2nd and 3rd PCs more clearly separated samples into patients and non-patients than the first one. This is the reason why we chose PCs 2 and 3, but not 1. On this plane, we selected 141 probes embedded in the PC scores as outliers according to a *χ*^2^ test with the *P* values adjusted by the Benjamini and Hochberg (BH) criterion^29^. Table 1 lists all 123 genes associated with the 141 probes.

**Figure 2:**
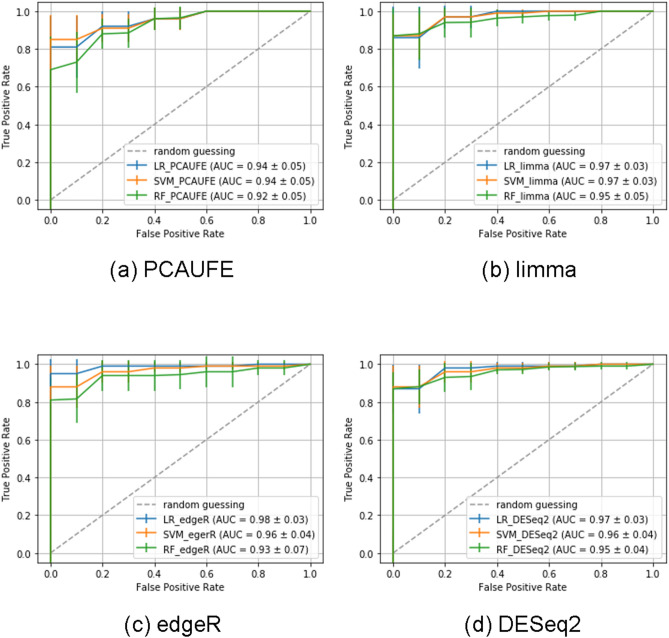
ROC curves of each classification model to predict COVID-19 patients or not based on the probes selected by (**a**) PCAUFE, (**b**) LIMMA, (**c**) edgeR, and (**d**) DESeq2, respectively. Note that since the number of probes respectively selected by LIMMA, edgeR, and DESeq2 were all above 4000, too much more than the samples, the probes with the smallest adjusted. *P* values were further restricted to be almost the same number as in PCAUFE, 141, for the explanatory variables. Using each classification model, we respectively performed fivefold cross-validation and reported the averages and standard deviations of the 5 runs.

To confirm that we successfully selected critical genes representing the relationship between samples, we built the model to predict the COVID-19 patients or not from only the 123 genes selected with PCAUFE. We used data set 2, which consisted of 100 COVID-19 patients and 26 non-COVID-19 ones, to calculate the area under the curve (AUC)^30^. We used logistic regression (LR)^31^, support vector machine (SVM)^32,33^, and random forest (RF)^34^ as classification models. Table S1 shows each hyperparameter of the three models. We performed 5-fold cross-validation by randomly shuffling the samples of data set 2 for the three classifiers. Figure 2(a) shows the receiver operating characteristic (ROC) curves^35,36^ of each model. As shown in this figure, the average AUC for each model was derived to be above 0.9. From these results, we could use the 123 selected genes for the prediction of the COVID-19 outcome.

**Table 1:** One hundred and twenty-three genes selected by PCAUFE. All of these genes were also selected by LIMMA.

ACTB	ACTG1	ADRBK1	AHNAK	ALAS2	ANXA1
ANXA2	APLP2	ARL4C	B2M	BTG1	BTG2
C1orf63	CCR7	CD14	CD163	CD69	CD74
CD83	CLU	COX1	CTSB	CTSS	CXCR4
CYFIP2	DDX3X	DDX5	DNAJB1	DUSP1	DUSP2
EEF1A1	EIF1	EIF4G2	ENO1	F13A1	FCN1
FLNA	FOS	FOSB	FTL	GAPDH	GLUL
GPR183	GRN	HBA1	HBA2	HBB	HLA-B
HLA-DPA1	HLA-DRA	HLA-DRB1	HLA-DRB5	HLA-E	HMHA1
HSP90B1	HSPA5	HSPA8	IFI27	IFITM3	IGJ
IL10RA	IL1B	IRF1	ISG15	ITGA2B	ITGB2
IVNS1ABP	JAK1	JUNB	JUND	KLF2	KLF6
LCK	LCP1	LOC100507709	LOC100507714	MAFB	MCL1
MX1	NFKBIA	NFKBIZ	NR4A1	NR4A2	PIK3IP1
PKM2	PLBD1	PNRC1	PPBP	PPP1R15A	PSAP
PTGER4	PTPRC	RGS2	RPL13	RPL3	RPS2
S100A12	S100A8	S100A9	SELL	SERPINA1	SF3B1
SH3BGRL3	SLC2A3	SORL1	SPARC	SRSF5	SRSF7
SUN2	TAGAP	TLN1	TMEM66	TNFAIP3	TNFRSF1B
TSC22D3	TUBA1A	TYMP	UBC	VCAN	YPEL5
ZFP36	ZFP36L2	ZNF331			

### Comparison with other gene selection methodologies

To confirm the robustness of our results, we performed gene selection with two other classical methods: significance analysis of microarrays (SAM)^37^ and linear models for microarray data (LIMMA)^38^. By applying both SAM and LIMMA to data set 1 (GSE152418), we identified genes associated with adjusted *P*-values below 0.01. We confirmed that the majority of the genes selected by PCAUFE were included among those selected by SAM and LIMMA. Every time SAM was applied, the selected genes changed. Thus, the results of SAM are not shown in this report. As for LIMMA, the selected probes included all of the genes selected by PCAUFE. It was noteworthy that PCAUFE could limit the candidate genes to a much smaller number than the common gene expression analysis tools could; for example, 18,458 probes were selected by LIMMA. By further limiting the 18,458 probes to almost the same number as in PCAUFE from the smallest adjusted *P*-values, we also performed the patient/non-patient classification from the genes selected by LIMMA using data set 2. The results using LR, SVM and RF are shown in Fig. 2(b). We confirmed that the classification performances of each model were comparable to those in PCAUFE.

To increase the robustness of our results, we also selected genes using some more recent R packages: edgeR^39^ and DESeq2^40^. As with SAM and LIMMA, by applying both edgeR and DESeq2 to data set 1, we identified genes associated with adjusted *P*-values below 0.01. The numbers of probes selected by edgeR and DESeq2 were 4452 and 5696, respectively. Thus, these methods selected much more genes than PCAUFE. The genes selected by edgeR and DESeq2 contained the 59 and 64 genes selected by PCAUFE, respectively. The common genes selected among the three methods were 57. For further comparison with PCAUFE, we conducted the classification analysis to predict whether the sample was the COVID-19 patient or not based on the genes selected by edgeR and DEseq2. In the classification analysis, we limited the same number of probes selected by each of edgeR and DEseq2 with the smallest adjusted *P*-values as in PCAUFE (i.e., 141), since both methods selected too many genes to use them for explanatory variables of the prediction models (i.e., 4452 and 5696). The numbers of genes associated with those probes were 111 for edgeR and 113 for DEseq2, respectively. Figures 2(c) and (d) shows the ROC curves of the patient-prediction models using the limited probes. Their AUCs were approximately equal to those in PCAUFE. As described above, we found that a smaller number of genes selected by PCAUFE than the other methods were significant in predicting the COVID-19 patients or not.

As the last part of this subsection, we conducted a weighted gene co-expression network analysis (WGCNA). Following the analytic procedure used in^41^ and^42^, we applied the WGCNA R package^43^ to data set 1. We here selected power of *β* = 7 as the soft threshold for constructing a scale-free network (Supplementary Fig. S2(A)). We then obtained 99 modules in the co-expression network as shown in Supplementary Fig. S2(B). These modules included 18882 probes, which were much more than those selected by PCAUFE (i.e., 141). Moreover, almost half (i.e., 58) of the genes selected by PCAUFE were contained in those by WGCNA. For reference, the protein-protein interaction (PPI) networks consisting of the genes that belonged to the top 3 modules with the smallest *P* values and the results of enrichment analyses for these genes are also shown in Supplementary Fig. S3 and Table S2, respectively.

As demonstrated above, we verified our approach, in which PCAUFE was adopted for gene selection, could narrow down the candidate genes more effectively than ordinary methods such as WGCNA, edgeR, and DEseq2.

## Discussion

In this study, we first identified 123 genes related to COVID-19 patients using PCAUFE. We justified the use of the linear dimensional reduction method by the following supplementary analysis: To compare with PCAUFE, we also applied *t*- distributed stochastic neighborhood embedding (t-SNE)^44^ and uniform manifold approximation and projection (UMAP)^45–47^, two typical nonlinear dimension reduction methods, to dataset 1 for gene selection. In the same manner with the PCAUFE algorithm, we tried to search the probes which passed the *χ*^2^ test with the *P* value adjusted by the BH criterion. As shown in Fig. S4, however, we found no probes with the adjusted *P* values less than 0.01 on the planes. The first algorithm of PCAUFE searches for principal components (i.e., axes) whose loadings statistically separate two groups such as patients and non-patients. Therefore, the outliers through the *χ*^2^ test in the second algorithm of PCAUFE could be regarded as the probes that were abnormally up-or down-regulated in some patients compared to non-patients. On the other hand, t-SNE and UMAP emphasize preserving the similarity of the probes as a distance when reducing a high-dimensional data set to two dimensions, the axes in the low-dimensional space do not always correspond to the two groups. Thus, it is not suitable for gene selection to naively use these nonlinear dimensional reduction methods because it does not mean that the probes located far from the origin in the low-dimensional space can serve as the biomarkers for the diagnosis of COVID-19.

Furthermore, it should be noted that the use of state-of-the-art deep learning techniques is not always successful in the gene expression analysis of a new type of disease such as COVID-19: We also applied a recently published method called single-cell Decomposition using Hierarchical Autoencoder (scDHA)^48^, that was described as reliably extracting representative information of each cell, to data set 1 for comparison to PCAUFE. Supplementary Figure S5 displays the scatter plot of samples of data set 1 in the dimension reduction space of scDHA. As shown in this figure, scDHA could not separate the COVID-19 patients from non-patients based on gene expression profiles of PBMCs at all. The reason is probably that this data set has too few samples and too many variables for training the autoencoder. PCAUFE is computationally less expensive than other methods because it only requires one application of PCA to a gene expression matrix in a peculiar way. Therefore, it has been successfully used to tackle a variety of gene selection problems (for detail, see e.g.,^49^). As described above, we consequently demonstrated the usefulness of this non-novel but powerful gene selection method for the data sets of gene expression profiles from COVID-19 patients and proposed a novel mechanism underlying the COVID-19 development.

We second implemented three independent models to classify COVID-19 patients and non-patients based on the 123 genes selected by PCAUFE: LR, SVM, and RF, and confirmed that all the models archived the high AUCs of over 90%. The reason why we did not use the state-of-art deep learning techniques for the classification model was that the sample size in the dataset used for the cross-validation (i.e., 126) was not large enough for the number of explanatory variables (i.e., 123). For example, it may be possible to build the classification model with deep learning by virtually increasing the number of samples as in^50^. Liu et al.^51^ used convolutional neural networks (CNNs) to predict Alzheimer’s patients based on the fMRI images of their hippocampus, but we did not use them because the explanatory variables in our patient prediction model were gene expression levels, in which the similarities could not be assumed between elements close in location as in images. However, as mentioned above, the statistical relevance of the genes selected by PCAUFE is already guaranteed because we found the conventional machine learning models had sufficient prediction accuracies.

To show the further robustness of our results, we also perform gene selection using data set 2 and clustering analysis to validate the separability by the selected genes using data set 1. The numbers of genes selected from data set 2 by PCAUFE, LIMMA, edgeR, and DESeq2 were 145, 7360, 4809, and 5018, respectively. LIMMA, edgeR, and DESeq2 respectively included the 79, 82, and 82 of 145 genes selected by PCAUFE using data set 2. On the other hand, the number of genes overlapping between the 123 and 145 genes selected by PCAUFE was 38. For the clustering analysis, we adopted an unsupervised learning model, UMAP, since data set 1 included only 34 samples, too few to train and test the supervised learning models using it. The results shown in Supplementary Fig. S6 indicate that the genes selected by PCAUFE could classify the COVID-19 patients or not as well as LIMMA, edgeR, and DESeq2. In the end, we got similar results even if we switched the data sets for gene selection and patient/non-patient classification.

Because we successfully confirmed the robustness of our results, we next investigated the biological reliability of the 123 selected genes. First, we uploaded the 123 genes to three enrichment analysis servers, GeneSetDB^52^, Metascape^53^, and TargetMine^54^, to compensate for the bias introduced by each individual enrichment. Multiple immune-related enrichments were detected. For example, Gene Ontology (GO) biological process (BP) terms GO:0019221 (cytokine-mediated signaling pathway), GO:0060333 (interferon-gamma-mediated signaling pathway), and GO:0060337 (type I interferon-mediated signaling pathway) were identified by all three servers. GO cellular component (CC) term GO:0042613 (MHC class II protein complex) was identified by GeneSetDB and TargetMine. Reactome pathways R-HSA-877300 (interferon gamma signaling), R-HSA-6785807 (Interleukin-4 and Interleukin-13 signaling), R-HSA-449147 (signaling by interleukins), and R-HSA-1280218 (adaptive immune system) were identified by Metascape and TargetMine (for more details, see supplemental S1_File).

Second, we confirmed biological validation of the identified genes by examining the interactions between them. Tight relationships between the genes would indicate that the gene selection was reliable, because single proteins rarely function without collaboration with other proteins. Thus, we uploaded the 123 genes to the STRING server^55^, which detected 659 protein–protein interactions among the products of these genes. Therefore, the 123 genes were also enriched for protein–protein interactions because of the functional collaborations between their products. These enrichment analyses suggested that PCAUFE could successfully identify a biologically feasible set of genes related to COVID-19.

**Figure 3:**
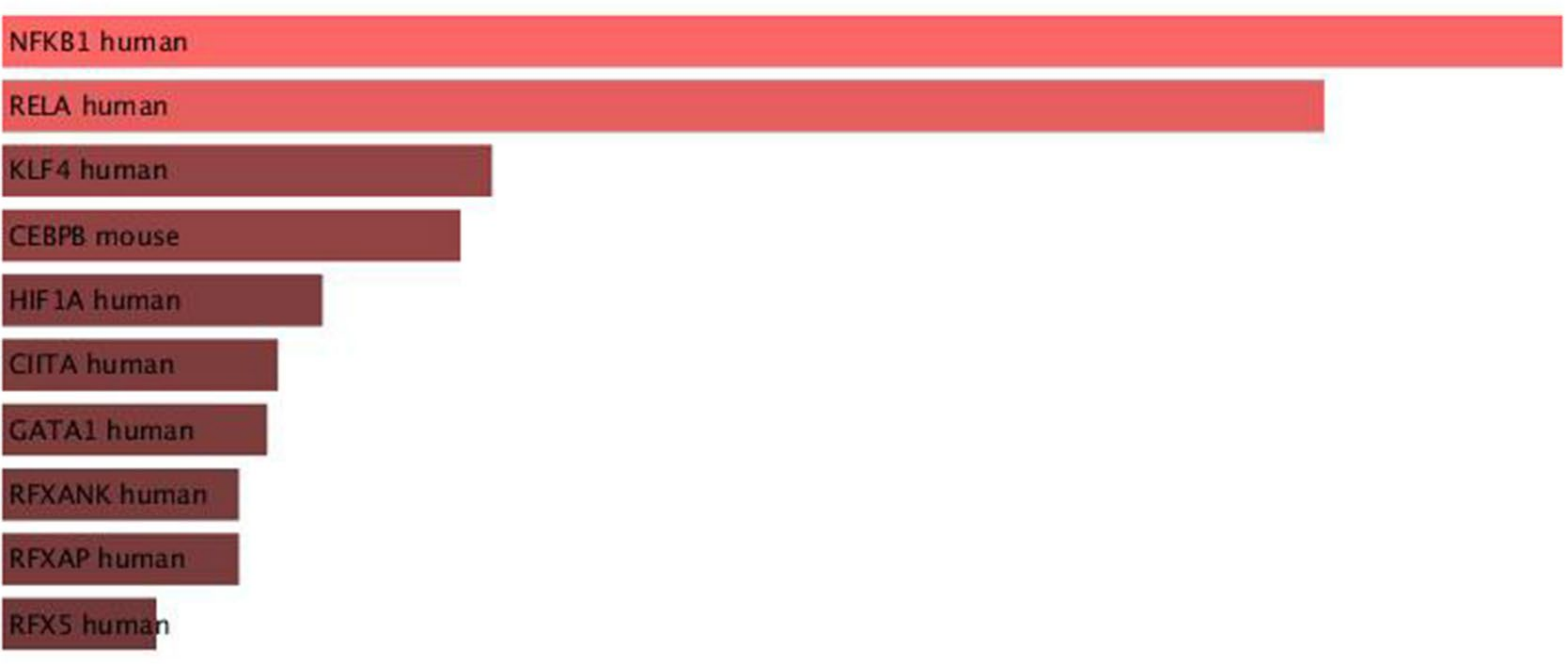
Bar graph of TRRUST Transcription Factors 2019. The graph visualizes the top ten enriched transcription factors of the genes selected by PCAUFE. The bars are colored and sorted according to their *P*-values.

To investigate the upstream transcription factors (TFs) that regulate the 123 genes selected by PCAUFE, we also uploaded them to Enrichr^56,57^, a multi-functional enrichment analysis server. Among the results given by Enrichr, NFKB1 and RELA had smaller adjusted *P*-values for “TRRUST Transcription Factors 2019,” as shown in Fig. 3. We also noticed the three highest-ranked TF bindings for “ENCODE TF ChIP-seq 2015”: NELFE, RELA, and KAT2A (for more details, see Fig. S7 in the supplemental S2_File).

**Figure 4:**
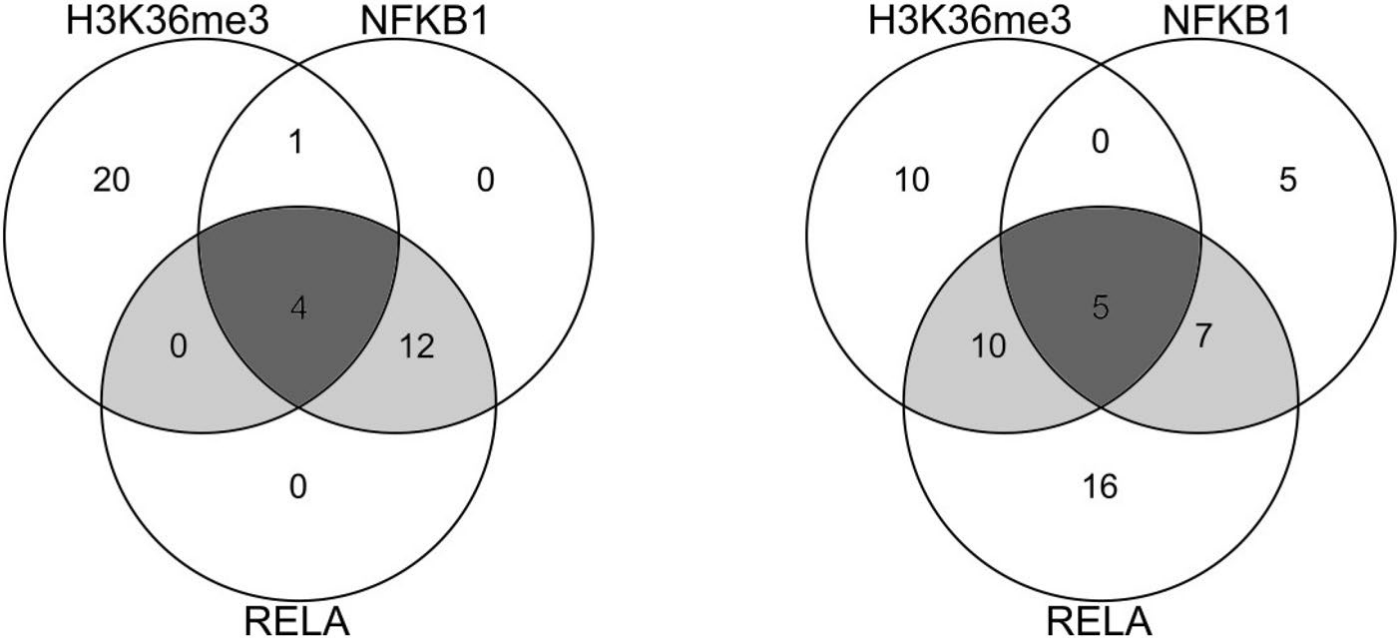
Venn diagrams of the enrichment of TF-binding sites and histone modifications for the 123 genes, as identified by Enrichr. The numbers in each diagram indicate the numbers of genes selected by PCAUFE and regulated by the TFs. Left: NFKB1, RELA (TRRUST Transcription Factors 2019), and H3K36me3_GM06990_hg19 (ENCODE Histone Modifications 2015); right: NFKB1 (TRRUST Transcription Factors 2019), RELA_GM12892_hg19 (ENCODE TF ChIP-seq 2015), and H3K36me3_GM06990_hg19 (ENCODE Histone Modifications 2015).

The 123 genes were also enriched for multiple histone modifications, and the results are listed in Table 2. Furthermore, as shown in Fig. 4, the genes associated with the histone modifications largely overlapped the TF target genes.

The nuclear factor-kappa B (NF-*κ*B) TFs play an evolutionarily conserved and critical role in the triggering and coordination of both innate and adaptive immune responses^58^. The NF-*κ*B family of transcription factors consists of five members: p50, p52, p65 (RelA), c-Rel, and RelB, which are encoded by NFKB1, NFKB2, RELA, REL, and RELB, respectively^59^. The primary mediator of canonical NF-*κ*B activity is the heterodimer RelA-p50, which consists of the RelA transcriptional activator and the nfkb1 protein p50^60,61^.

Nakshatri et al.^62^ suggested that NF-*κ*B activity is suppressed by H3K36me3, which is consistent with the observed enrichment of NFKB1- and RELA-binding sites in these 123 genes. Many studies have also reported that the expression levels of genes associated with immune signaling are downregulated in naso/oropharyngeal swabs and peripheral blood mononuclear cells (PBMCs) in patients with COVID-19. Mick et al.^63^ showed that COVID-19 is characterized by a diminished innate immune response, with reduced expression of genes involved in toll-like receptor and interleukin signaling, chemokine binding, neutrophil degranulation, and interactions with lymphoid cells, as compared to other viral acute respiratory illnesses. Meckiff et al.^64^ showed that SARS-CoV-2-reactive CD4^+^ T cells express significantly lower levels of immune-related transcripts as compared to influenza-reactive cells. Ouyang et al.^65^ reported that the genes that are underexpressed in severe cases mainly involve Th17-cell differentiation, cytokine-mediated signaling pathways, and T-cell activation. Li et al.^66^ reported that proteins mediating T-cell receptor signaling are downregulated in severe COVID-19 PBMCs.

**Figure 5:**
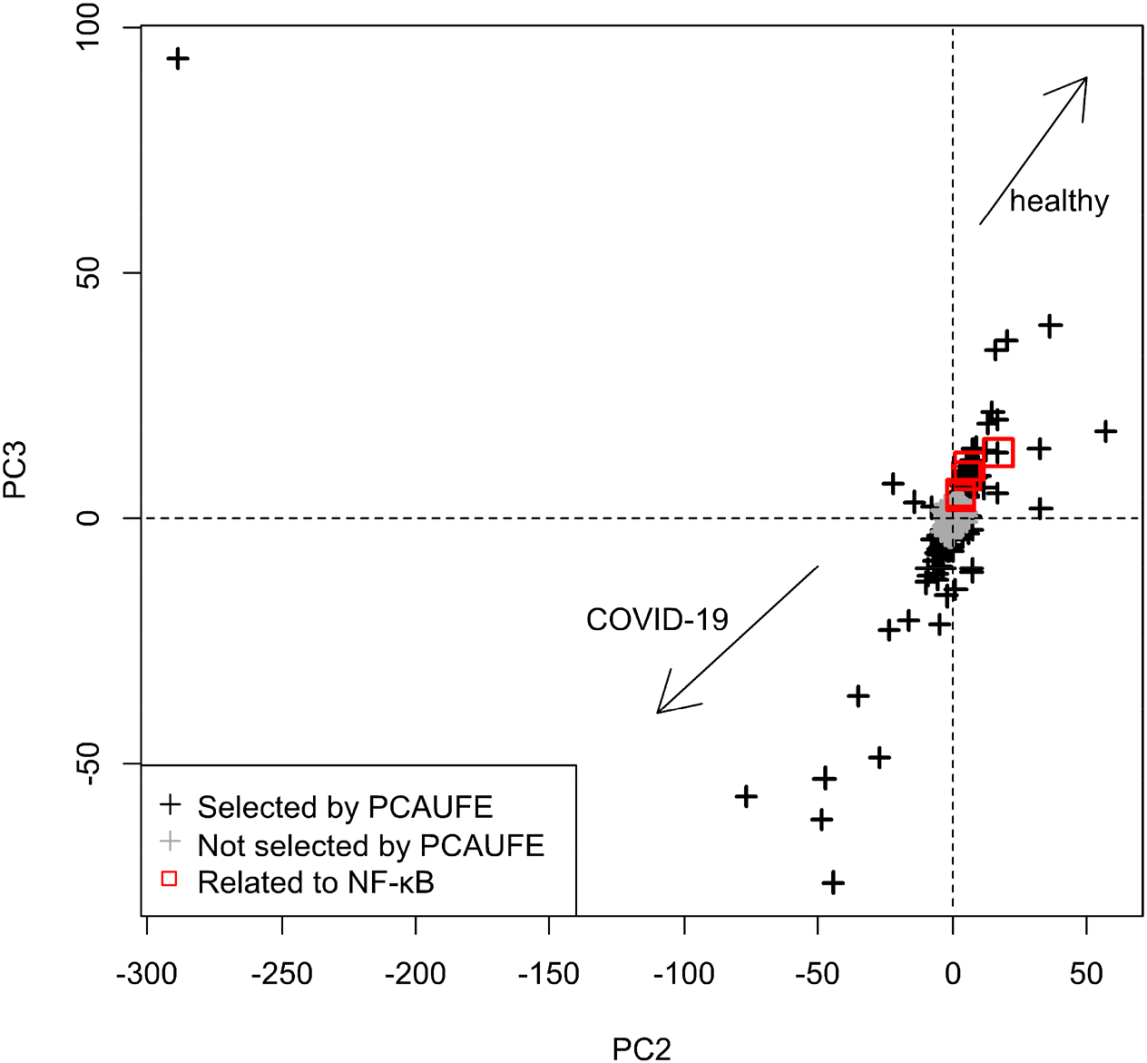
Scatter plot of the PC2 and PC3 scores for data set 1. The black crosses represent the probes selected by PCAUFE, while the gray crosses represent unselected probes. The red squares are associated with the five overlapping genes in Fig. 4.

To investigate the expression variation of the five overlapping genes in Fig. 4, we confirmed the PC2 and PC3 scores for data set 1 via the scatter plot shown in Fig. 5. The probes that were negatively located for both PC2 and PC3 were mainly upregulated for COVID-19. On the other hand, the probes that were positively located for both PCs were mainly downregulated for COVID-19. As shown by the red squares in Fig. 5, the overlapping genes in Fig. 4 were positively located for both PCs. Therefore, those overlapping genes were downregulated for COVID-19. These analysis results are consistent with the above references^62–66^.

NF-*κ*B is the subject of much active research among pharmaceutical companies as a target for anti-cancer therapy^67^. Abnormal expressions of NFKB1 and RELA are mediated through mRNA modifications^68^. The recent progress in N4- Acetylcytidine (N4A) on RNA expression is also playing key role on the cancer development^69^. Duan J et al.^70^ reported that N4A, a nucleoside metabolite, activated microglia and sustained NLRP3 inflammasome activation by inducing HMGB1 signaling. Released HMGB1 through N4A activated NF-*κ*B and induced NLRP3 expression^70^. NLRP3 inflammasome appropriately activated and enabled to release mature IL-1*β*^71–73^. IL-1*β* is an important mediator of the inflammatory response, and is involved in a variety of cellular activities, including cell proliferation, differentiation, and apoptosis^74,75^.

Although we identified the 123 genes and the upstream TFs related to COVID-19 by using PCAUFE and enrichment analyses, we have yet assessed whether these genes have the potential causal effects on the COVID-19 development. Mendelian randomization (MR)^76–80^ approach has been widely used to investigate causality between genes and disease outcomes. For example, Zhang et al.^79^ investigated the causal relationships between PTSD and the depressive phenotypes using an MR approach. In another of their studies^80^, The results of MR analysis indicate that genetic variation mediates the causal influences of neuroticism on mental health and cardiovascular diseases. This method uses genetic polymorphism information as an operating variable, but unfortunately, the data sets we used do not include that information. Moreover, polymorphisms may have several phenotypic effects associated with the disease. Thus, we leave the application of the MR approach to the gene expression profiles from COVID-19 patients as future work.

In conclusion, we selected 123 COVID-19-related genes by applying PCAUFE to the gene expression levels of PBMCs from COVID-19 patients and healthy subjects. Then, by enrichment analysis, we identified the transcription factors and histone modifications that regulate the expression of these genes. Four transcription factors, NELFE, RELA, KAT2A and NFKB1, and a histone modification, H3K36me3, may be involved in the expression of the 123 genes. NFKB1, RELA, and H3K36me3 were found to overlap in the genes regulating expression. These two transcription factors are associated with NF-*κ*B, and H3K36me3 may repress it. In fact, when we compared the expression levels of the genes duplicated in NFKB1, RELA, and H3K36me3 in GSE152418 between the COVID-19 patients and healthy subjects, we observed a decrease in expression levels in the COVID-19 patients. These results suggest that canonical NF-*κ*B activity is suppressed by H3K36me3 in the PBMCs of COVID-19 patients.

**Table 2:** Enriched histone modifications detected by Enrichr (ENCODE Histone Modifications 2015) for the 123 selected genes. Only those with adjusted *P*-values below 0.05 are listed here.

Rank	Histone modification	*P* value	adjusted *P* value	combined score
1	H3K36me3 Caco-2 hg19	1.93E−14	7.94 E−12	282.29
2	H3K36me3 kidney epithelial cell hg19	7.73 E−10	1.59 E−07	206.28
3	H3K36me3 bronchial epithelial cell hg19	1.26 E−09	1.73 E−07	65.42
4	H3K36me3 splenic B cell mm9	7.07 E−09	5.83 E−07	53.40
5	H3K36me3 thymus mm9	6.05 E−09	6.23 E−07	67.27
6	H3K36me3 spleen mm9	2.57 E−08	1.76 E−06	48.31
7	H3K36me3 GM06990 hg19	1.49 E−07	8.75 E−06	50.92
8	H3K36me3 BJ hg19	2.98 E−07	1.53 E−05	46.92
9	H3K36me3 kidney mm9	3.08 E−06	1.41 E−04	38.25
10	H3K36me3 SK-N-SH hg19	7.04 E−06	2.90 E−04	43.66
11	H4K20me1 skeletal muscle myoblast hg19	8.88 E−06	3.32 E−04	27.43
12	H3K36me3 myocyte mm9	1.56 E−05	5.36 E−04	68.63
13	H3K36me3 H7 hg19	1.76 E−05	5.57 E−04	25.97
14	H3K36me3 MCF-7 hg19	3.85 E−05	1.13 E−03	26.07
15	H3K36me3 C2C12 mm9	4.34 E−05	1.19 E−03	44.15
16	H3K36me3 small intestine mm9	1.77 E−04	4.56 E−03	18.26
17	H4K20me1 fibroblast of lung hg19	4.39 E−04	1.06 E−02	15.72
18	H3K36me3 cardiac mesoderm hg19	5.37 E−04	1.23 E−02	14.12
19	H3K36me3 CD14-positive monocyte hg19	1.04 E−03	2.14 E−02	13.41
20	H4K20me1 GM12878 hg19	1.04 E−03	2.25 E−02	13.41
21	H3K36me3 CH12.LX mm9	1.63 E−03	3.20 E−02	10.10
22	H4K20me1 keratinocyte hg19	2.34 E−03	4.19 E−02	11.33
23	H4K20me1 mammary epithelial cell hg19	2.34 E−03	4.38 E−02	11.33

## Methods

### Gene expression profiles

Two *in vivo* gene expression data sets, GSE152418^28^ and GSE157103^81^, were downloaded from Gene Expression Omnibus^82^. Hereafter, we denote these as data sets 1 and 2, respectively. PCAUFE was applied to data set 1, which described the expression level of each kind of mRNA in each subject’s PBMCs. The number of probes was 60,683. Data set 2 was then used to confirm the statistical validity of the genes selected by PCAUFE. This data set also described the expression level of each gene in each subject’s PBMCs. The data included both COVID-19 patients and non-COVID-19 patients who suffered from acute respiratory distress syndrome (ARDS) that was not associated with SARS-CoV-2. The number of genes was 19,472. The expression level of each gene *i* (= 1*,* 2,…, *N*) was standardized for PCAUFE, i.e., we set $\frac{1}{N}\sum_{i} x_{ij} = 0$ and $\frac{1}{N}\sum_{i} x_{ij}^{2} = 0$. For the details of the samples included in these gene expression profiles, see Table S3 in the supplemental S2_File.

### PCAUFE

The following briefly explains the PCAUFE procedure used in this study (for more details, see^9,12,17,27,49^). Let *x*_*i j*_ be the expression of the *i*-th mRNA probe of the *j*th sample, and let $\frac{1}{N}\sum_{i} x_{ij} = 0$ and $\frac{1}{N}\sum_{i} x_{ij}^{2} = 0$, where *N* is the number of m-RNA probes. First, we applied PCA to the dataset whose rows and columns were genes and samples, respectively. In contrast to the usual use of PCA, where samples are embedded, the genes were embedded in this implementation. By using a *t*-test, we specified two principal components (PCs) whose loadings statistically differentiated the patients from healthy control samples in order from the smallest *P*-value. Note that, unlike ordinary PCA, this operation does not guarantee that the first two PCs will be selected. Second, by using a *χ*^2^ test with the *P* values adjusted by the BH criterion^29^, we identified outlier PC scores (i.e., genes associated with the adjusted *P*-values less than 0.01) along with the specified PCs as candidates for the disease-related genes. Note that the PC scores in PCAUFE were associated with features (i.e., mRNA probes), not with samples, in contrast to the ordinary usage of PCA.

### Patient/non-patient classification models

To verify the genes selected by PCAUFE were useful for the diagnosis of COVID-19 patients, we performed the patient/non- patient classification based on the selected genes using three standard prediction models: logistic regression (LR^31^), support vector machine (SVM^32,33^) and random forest (RF^34^). For the details of each hyperparameter of the three models, see Supplementary Table S1. The objective variable was given the value 0 or 1 for each sample depending on a non-COVID-19 or a COVID-19 patient, respectively. The explanatory variables were given the gene expressions of the probes associated with the genes selected by PCAUFE, edgeR, or DESeq2. We randomly allocated 80% of data set 2 to the training set and the remains to the test one. Receiver operating characteristic (ROC) curves of each model were drawn to calculate the area under the curves (AUCs).

## Supplementary Information

Supplementary Information.

Supplementary Information.
