# Supplementary material for: PCA-based unsupervised feature extraction for gene expression analysis of COVID-19 patients
Source: Sci Rep. 2021 Aug 30;11:17351. doi: 10.1038/s41598-021-95698-w (PMC8403676; doi:10.1038/s41598-021-95698-w)
Supplement: Supplementary file 1 — Supplementary Information. [file 41598_2021_95698_MOESM1_ESM.pdf]

PCA-based unsupervised feature extraction for gene expression analysis of COVID-19 patients

Kota Fujisawa[1, *], Mamoru Shimo[2], Y-h. Taguchi[3], Shinya Ikematsu[4], Ryota Miyata[5, *]

[1] School of Life Science and Technology, Tokyo Institute of Technology, Tokyo 152-8550, Japan

[2] Graduate School of Engineering and Science, University of the Ryukyus, Okinawa 903-0213, Japan

[3] Chuo University, Department of Physics, Tokyo 112-8551, Japan

[4] National Institute of Technology, Okinawa College, Department of Bioresources Engineering, Okinawa 905-2192, Japan

[5] University of the Ryukyus, Faculty of Engineering, Okinawa 903-0213, Japan

* fujisawa.k.ab@m.titech.ac.jp (K. F.) and miyata26@tec.u-ryukyu.ac.jp (R. M.)

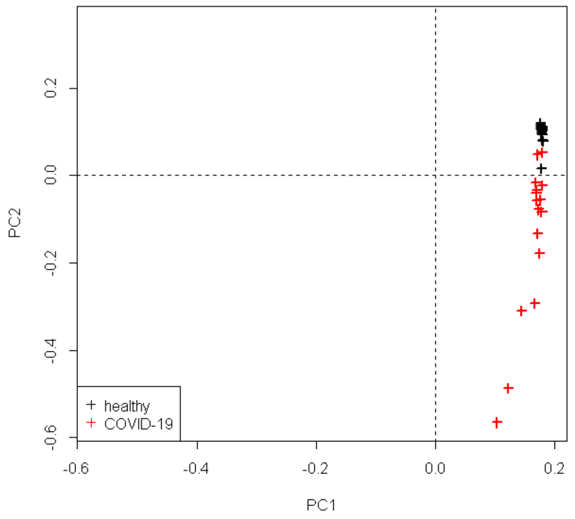

(a) PC1 and PC2

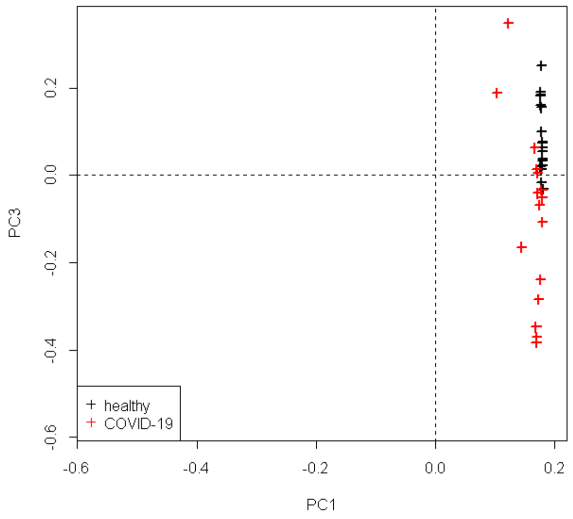

(b) PC1 and PC3

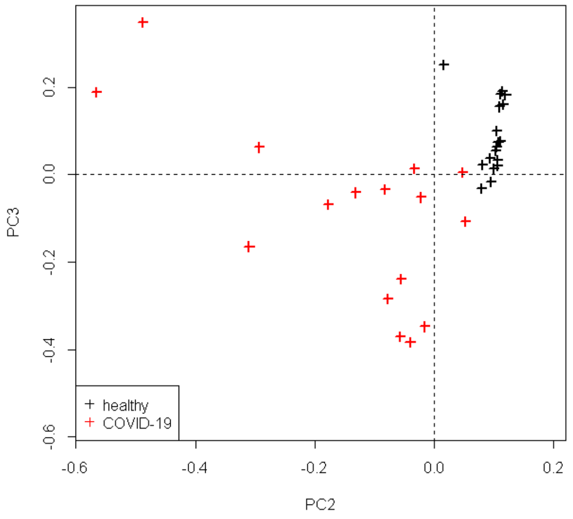

(c) PC2 and PC3

Figure S1. Scatter plots of the PC loadings for data set 1. Note that in the PCAUFE algorithm, samples were embedded in the PC loadings, not in the PC scores.

(A)

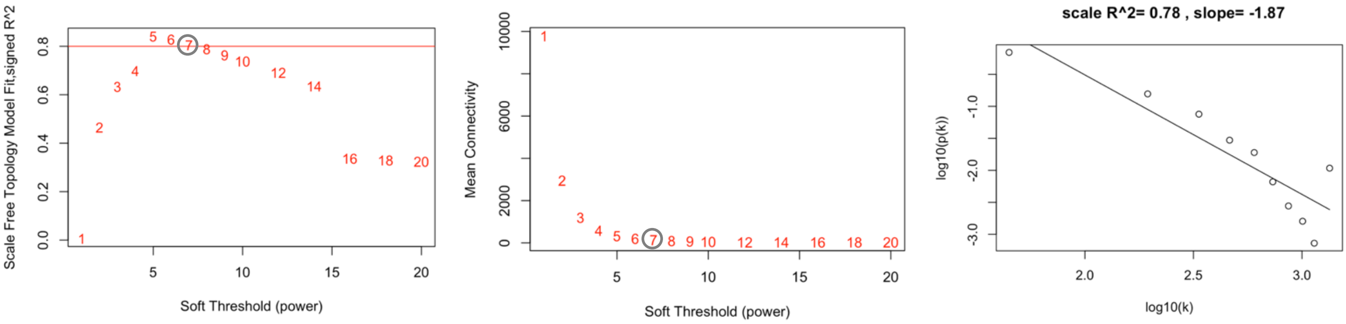

(B)

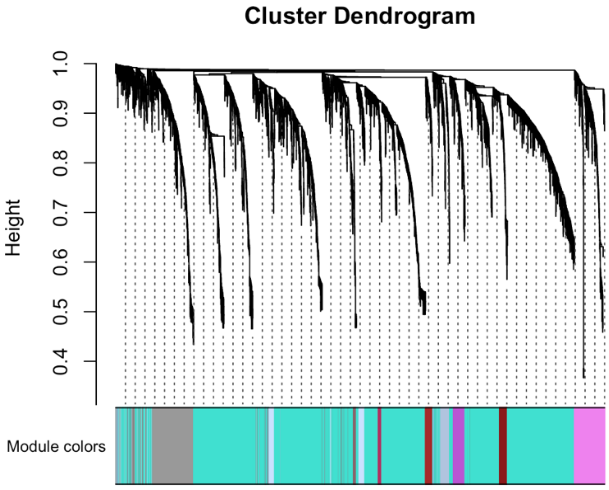

Figure S2. Parameter analysis of inferred co-expression network and modules using the WGCNA R package. (A) Determination of the scaling factor based on the scale-free topology criterion (red solid line in the left-sided figure) and verification of β = 7 satisfying the scale-free topology (black solid line in the right-sided figure). (B) Hierarchical clustering of genes in significant modules. The 99 colors are assigned to each module by the dynamic tree cut algorithm.

(A) Black Module

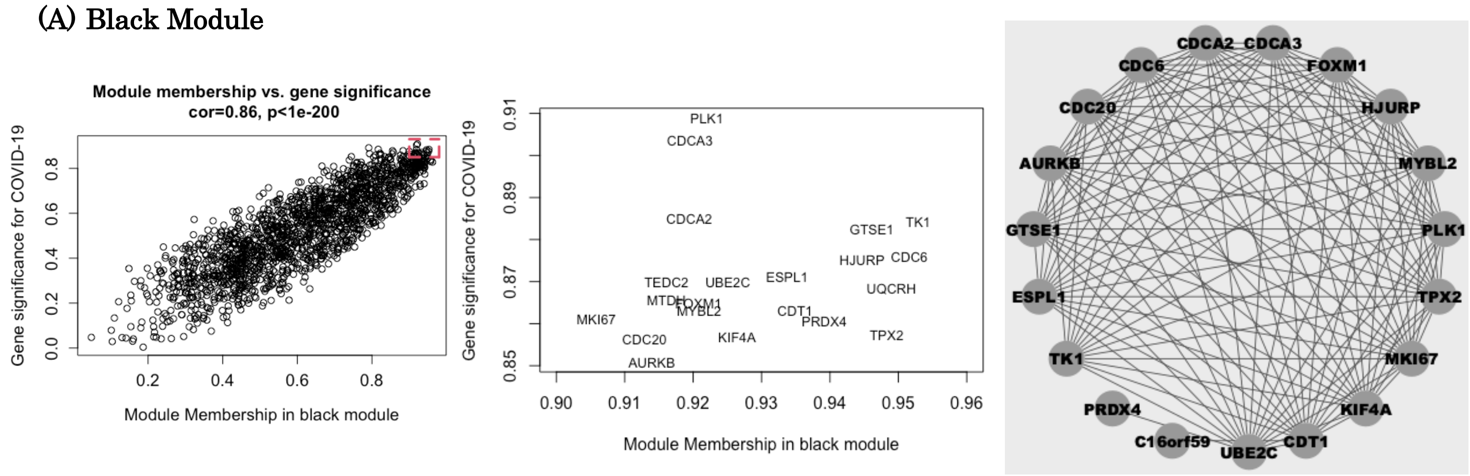

(B) Midnight-blue Module

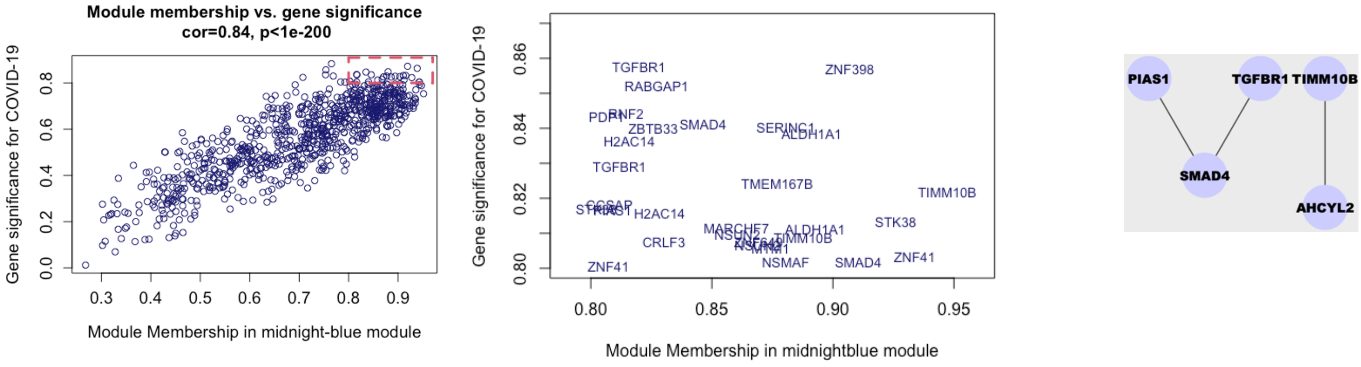

(C) Blue Module

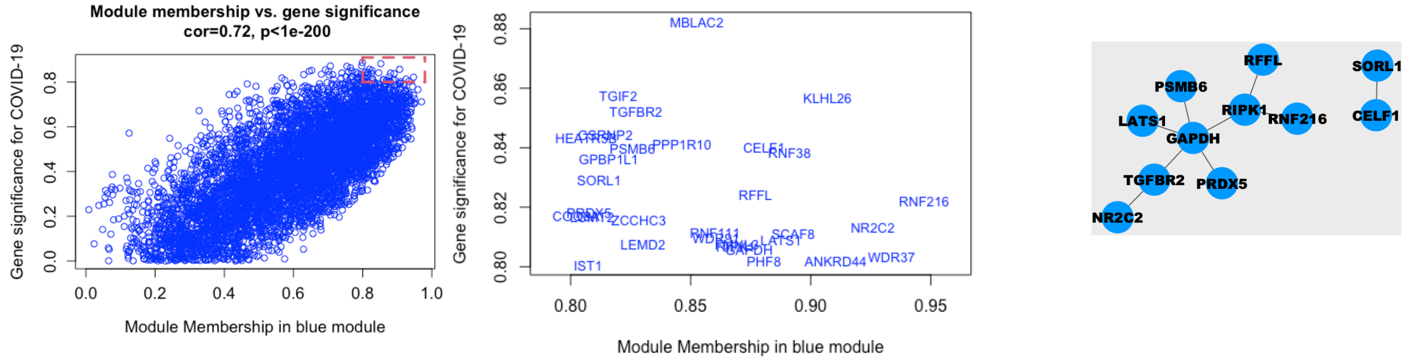

Figure S3. Module_membership-gene_significance correlation analyses and the protein-protein interaction (PPI) network analyses in the (A) black, (B) midnight-blue, and (C) blue modules, which had the top 3 smallest *P*-values among the 99 modules shown in Supplementary Fig. S3. (Left-sided column) Scatter plots of the correlation between gene significance (GS) and module membership (MM). (Middle column) The top 20-30 genes with high GS and MM, corresponding to those surrounded by the red dotted line shown in the figures in the left-sided column. (Right-sided column) PPI networks composed of genes with high GS and MM shown in the figures in the middle column.

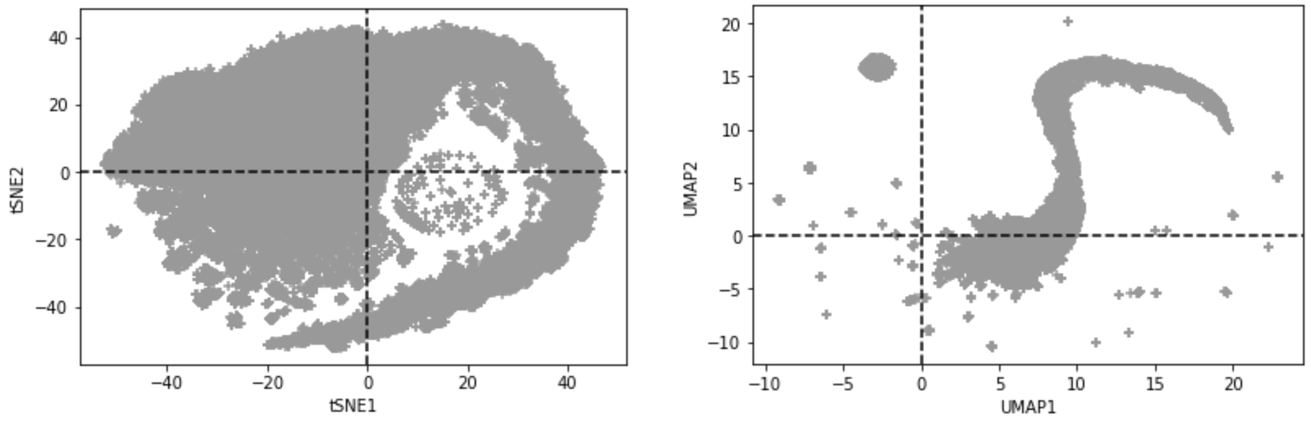

(a) t-SNE (b) UMAP

Figure S4. Scatter plot of the two typical nonlinear dimension reduction methods for data set 1. No probes were selected by the chi square test.

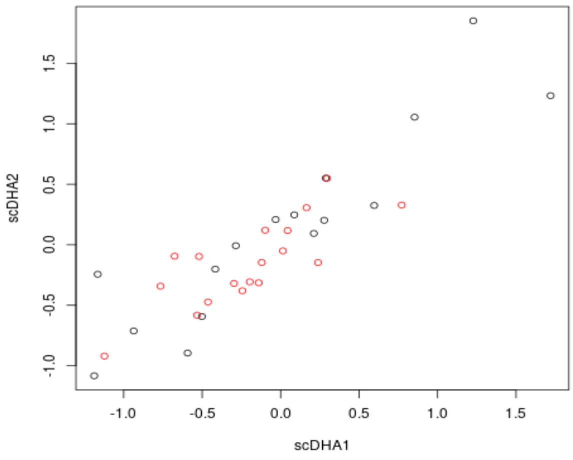

Figure S5. The scDHA visualization of data set 1. The black-lined circles represent the COVID-19 patients, and the red-lined ones do the non-patients.

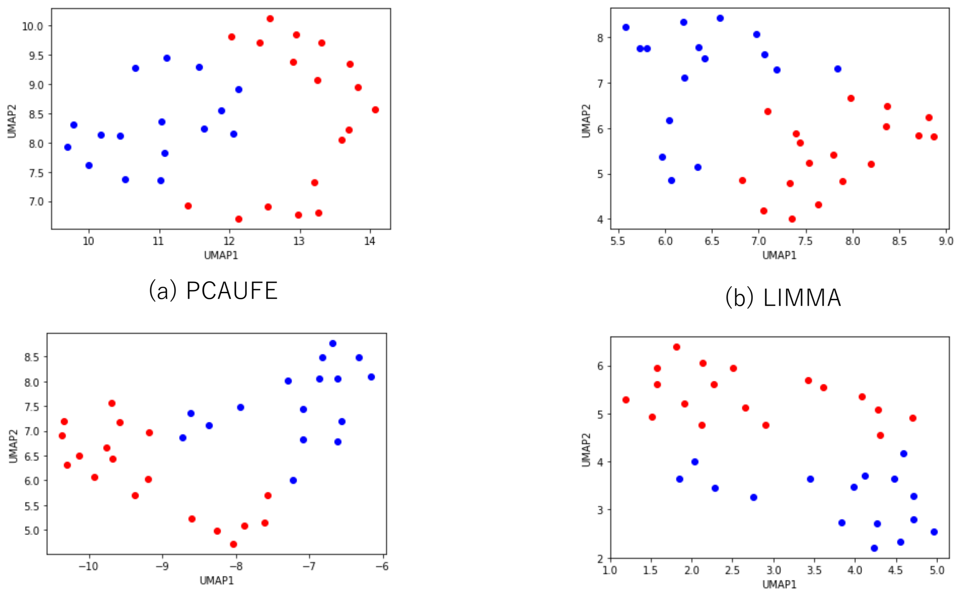

(a) PCAUFE (b) LIMMA

(c) edgeR (d) DESeq2

Figure S6. The UMAP visualization of data set 1 using only the genes respectively selected from data set 2 by each method. The blue dots represent the COVID-19 patients, and the red ones do the non-patients.

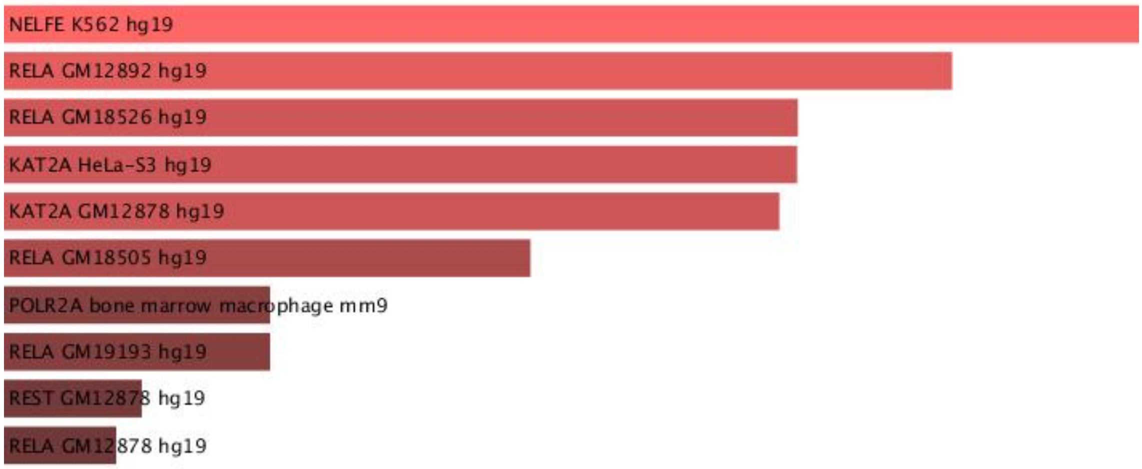

Figure S7. ENCODE TF ChIP-seq 2015. The graph visualizes the top ten enriched transcription factors of the genes selected by PCAUFE. The bars are colored and sorted according to their *P*-values.

Table S1. Each hyperparameter of classification models to predict COVID-19 patients or not based on the 123 genes selected by PCAUFE. For the other parameters of RF, we adopted the default settings of scikit-learn 0.19.1[1]. These hyperparameters were also used in the classification analysis with edgeR and DEseq2 shown in Fig. S2.

(a) LR

C	penalty
1.0	l2

(b) SVM

C	gamma	kernel
1.0	0.0001	rbf

(c) RF

max_features	criterion	n_estimator
auto	gini	20

Table S2. Ontologies of genes assigned to the black, midnight-blue, and blue modules shown in Supplementary Fig. S4. The GO enrichment analyses were performed using GeneSetDB.

(A) Black Module

Category	GO term	Count of genes	FDR
BP	mitotic cell cycle	111	2.60E-51
BP	respiratory electron transport chain	49	3.80E-34
BP	cell division	77	3.70E-27
BP	cell cycle checkpoint	52	1.00E-26
BP	G1/S transition of mitotic cell cycle	54	5.30E-26
CC	mitochondrial inner membrane	75	2.40E-23
BP	S phase of mitotic cell cycle	45	4.00E-23
BP	M/G1 transition of mitotic cell cycle	38	8.20E-23
BP	M phase of mitotic cell cycle	39	5.00E-21
CC	respiratory chain	27	3.80E-20

(B) Midnight-blue Module

Category	GO term	Count of genes	FDR
BP	protein transport	44	8.90E-07
BP	vesicle-mediated transport	21	2.50E-03
CC	late endosome	11	2.20E-02
CC	Golgi membrane	31	5.40E-02
BP	intracellular protein transport	18	5.40E-02
MF	ligase activity	28	5.40E-02
BP	activation of MAPKK activity	8	5.40E-02
BP	cellular membrane organization	11	7.20E-02
BP	epidermal growth factor receptor signaling pathway	13	9.40E-02
BP	endosome transport	7	1.10E-01

(C) Blue Module

Category	GO term	Count of genes	FDR
MF	helicase activity	55	3.50E-07
BP	regulation of transcription from RNA polymerase II promoter	85	3.10E-06
BP	negative regulation of transcription, DNA-dependent	127	1.30E-05
BP	chromatin modification	79	1.10E-04
MF	nucleic acid binding	123	4.30E-04
MF	transcription coactivator activity	76	5.20E-04
MF	protein kinase activity	72	6.70E-04
MF	ubiquitin-protein ligase activity	81	7.00E-04
CC	nuclear speck	49	4.30E-03
MF	protein serine/threonine kinase activity	105	4.70E-03

Table S3. List of samples included in data sets 1 and 2. Upper: data set 1 (GSE152418[1]); lower: data set 2 (GSE157103[2]). For data set 2, we defined a patient with a relatively severe symptom as a severe patient (SP).

Data set 1 (GSE152418[1])

Label	Severity	Samples	Total
COVID-19	ICU patient (IP)	4	16
	Severe patient (SP)	8	
	Moderate patient (MP)	4	
healthy	Convalescent patient (CP)	1	18
	Healthy control (HC)	17	

Data set 2 (GSE157103[2])

COVID-19	SP	8	100
	MP	92	
Non-COVID-19	SP	1	26
	MP	25	

Reference

1. https://scikit-learn.org/stable/
