# Supplementary material for: PCA-based unsupervised feature extraction for gene expression analysis of COVID-19 patients
Source: Sci Rep. 2021 Aug 30;11:17351. doi: 10.1038/s41598-021-95698-w (PMC8403676; doi:10.1038/s41598-021-95698-w)
Supplement: Supplementary file 2 — Supplementary Information. [file 41598_2021_95698_MOESM2_ESM.pdf]

File S1 (a) TargetMine: Gene Ontology (Biological Process)

Identifier	Name	Foreground	Background	p-value
GO:0002252	immune effector process	33	897	5.65E-15
GO:0001775	cell activation	34	996	6.93E-15
GO:0002376	immune system process	46	2120	1.47E-14
GO:0045321	leukocyte activation	31	881	8.59E-14
GO:0006955	immune response	38	1492	2.28E-13
GO:0002366	leukocyte activation involved in immune response	24	558	6.63E-12
GO:0002263	cell activation involved in immune response	24	561	6.63E-12
GO:0034097	response to cytokine	26	760	6.35E-11
GO:0071345	cellular response to cytokine stimulus	24	643	1.05E-10
GO:0019221	cytokine-mediated signaling pathway	23	597	1.81E-10
GO:0070887	cellular response to chemical stimulus	40	2101	2.28E-10
GO:0010033	response to organic substance	38	2014	1.65E-09
GO:0016192	vesicle-mediated transport	34	1618	1.99E-09
GO:0006952	defense response	28	1066	1.99E-09
GO:0042221	response to chemical	44	2751	2.26E-09
GO:0045055	regulated exocytosis	22	626	2.69E-09
GO:0006887	exocytosis	23	709	3.77E-09
GO:0006810	transport	51	3869	1.42E-08
GO:0071310	cellular response to organic substance	33	1651	1.43E-08
GO:0007165	signal transduction	54	4364	2.03E-08
GO:0006950	response to stress	42	2752	3.72E-08
GO:0051234	establishment of localization	51	3994	4.13E-08
GO:0050789	regulation of biological process	78	9301	4.46E-08
GO:0044419	biological process involved in interspecies interaction between organisms	28	1251	5.01E-08
GO:0009605	response to external stimulus	33	1747	5.01E-08
GO:0051179	localization	58	5172	7.11E-08
GO:0043312	neutrophil degranulation	17	423	8.91E-08
GO:0002283	neutrophil activation involved in immune response	17	426	9.58E-08
GO:0002446	neutrophil mediated immunity	17	430	1.07E-07
GO:0042119	neutrophil activation	17	433	1.15E-07

GO:0051649	establishment of localization in cell	37	2273	1.24E-07
GO:0036230	granulocyte activation	17	437	1.24E-07
GO:0002274	myeloid leukocyte activation	18	516	1.85E-07
GO:0002443	leukocyte mediated immunity	19	584	1.85E-07
GO:0043299	leukocyte degranulation	17	451	1.85E-07
GO:0046903	secretion	25	1067	1.89E-07
GO:0002275	myeloid cell activation involved in immune response	17	455	2.00E-07
GO:0051641	cellular localization	41	2826	2.10E-07
GO:0002444	myeloid leukocyte mediated immunity	17	458	2.10E-07
GO:0065007	biological regulation	79	9884	2.17E-07
GO:0032940	secretion by cell	24	1011	3.14E-07
GO:0007154	cell communication	54	4752	3.36E-07
GO:0023052	signaling	54	4762	3.49E-07
GO:0009607	response to biotic stimulus	23	932	3.49E-07
GO:0002224	toll-like receptor signaling pathway	10	122	4.98E-07
GO:0140352	export from cell	24	1053	6.33E-07
GO:0002221	pattern recognition receptor signaling pathway	11	169	8.10E-07
GO:0001816	cytokine production	17	513	9.52E-07
GO:0043207	response to external biotic stimulus	22	907	0.000001
GO:0051707	response to other organism	22	907	0.000001
GO:0051716	cellular response to stimulus	53	4743	0.000001
GO:0097530	granulocyte migration	9	99	0.0000012
GO:0050896	response to stimulus	63	6499	0.0000012
GO:0030097	hemopoiesis	16	460	0.0000013
GO:0016477	cell migration	23	1022	0.0000016
GO:0048534	hematopoietic or lymphoid organ development	16	472	0.0000018
GO:0002521	leukocyte differentiation	13	297	0.0000027
GO:0002682	regulation of immune system process	23	1053	0.0000027
GO:0050794	regulation of cellular process	73	8757	0.0000034
GO:0050729	positive regulation of inflammatory response	8	81	0.000004
GO:0007166	cell surface receptor signaling pathway	33	2146	0.0000043
GO:0019222	regulation of metabolic process	56	5449	0.0000043

GO:0006954	inflammatory response	14	376	0.0000049
GO:0071621	granulocyte chemotaxis	8	85	0.0000055
GO:0008219	cell death	26	1401	0.0000059
GO:0002694	regulation of leukocyte activation	13	324	0.0000066
GO:0030217	T cell differentiation	9	128	0.0000089
GO:0042110	T cell activation	12	279	0.0000107
GO:0007159	leukocyte cell-cell adhesion	11	225	0.0000108
GO:0046649	lymphocyte activation	14	405	0.0000113
GO:1903131	mononuclear cell differentiation	11	227	0.0000115
GO:0048870	cell motility	23	1162	0.0000135
GO:0098609	cell-cell adhesion	16	558	0.0000146
GO:0050865	regulation of cell activation	13	350	0.0000146
GO:0060255	regulation of macromolecule metabolic process	52	4986	0.000016
GO:0010468	regulation of gene expression	45	3912	0.0000172
GO:0002286	T cell activation involved in immune response	7	69	0.0000209
GO:0048584	positive regulation of response to stimulus	27	1614	0.0000209
GO:0098542	defense response to other organism	18	737	0.0000209
GO:0009893	positive regulation of metabolic process	36	2695	0.0000213
GO:0030098	lymphocyte differentiation	10	194	0.0000238
GO:0030593	neutrophil chemotaxis	7	72	0.000027
GO:0097529	myeloid leukocyte migration	9	151	0.0000298
GO:0050900	leukocyte migration	12	313	0.0000303
GO:0048518	positive regulation of biological process	49	4615	0.0000313
GO:0050727	regulation of inflammatory response	10	201	0.0000313
GO:0030595	leukocyte chemotaxis	9	156	0.0000376
GO:0051249	regulation of lymphocyte activation	11	263	0.0000413
GO:0040011	locomotion	23	1284	0.000066
GO:0060326	cell chemotaxis	10	220	0.0000688
GO:0001817	regulation of cytokine production	14	480	0.0000692
GO:0002250	adaptive immune response	9	169	0.0000694
GO:1990266	neutrophil migration	7	84	0.0000694
GO:0010604	positive regulation of macromolecule metabolic process	33	2453	0.0000721
GO:1901700	response to oxygen-containing compound	18	815	0.0000768
GO:0044403	biological process involved in symbiotic interaction	14	488	0.00008
GO:0046631	alpha-beta T cell activation	7	87	0.0000846
GO:0045087	innate immune response	15	584	0.0001193
GO:0050776	regulation of immune response	16	668	0.0001219

GO:0048583	regulation of response to stimulus	37	3048	0.0001241
GO:0016032	viral process	13	436	0.0001316
GO:1903706	regulation of hemopoiesis	10	240	0.0001333
GO:0006915	apoptotic process	21	1140	0.0001333
GO:0051246	regulation of protein metabolic process	27	1803	0.0001486
GO:0035710	CD4-positive, alpha-beta T cell activation	6	62	0.000175
GO:0009892	negative regulation of metabolic process	31	2313	0.0001881
GO:0010467	gene expression	48	4747	0.0001971
GO:0048522	positive regulation of cellular process	44	4135	0.0002088
GO:0065009	regulation of molecular function	27	1843	0.0002183
GO:0006898	receptor-mediated endocytosis	9	198	0.0002206
GO:0051674	localization of cell	16	705	0.0002206
GO:0042093	T-helper cell differentiation	5	37	0.0002441
GO:0002576	platelet degranulation	7	104	0.0002445
GO:0002696	positive regulation of leukocyte activation	9	202	0.0002512
GO:0002294	CD4-positive, alpha-beta T cell differentiation involved in immune response	5	38	0.0002726
GO:0071702	organic substance transport	27	1879	0.0002989
GO:0002287	alpha-beta T cell activation involved in immune response	5	39	0.0003034
GO:0002293	alpha-beta T cell differentiation involved in immune response	5	39	0.0003034
GO:1902105	regulation of leukocyte differentiation	8	155	0.0003097
GO:0006928	movement of cell or subcellular component	24	1539	0.000312
GO:0050867	positive regulation of cell activation	9	209	0.0003134
GO:0002285	lymphocyte activation involved in immune response	7	110	0.0003301
GO:0010941	regulation of cell death	19	1028	0.0003924
GO:0006811	ion transport	32	2548	0.000415
GO:0002292	T cell differentiation involved in immune response	5	42	0.000415
GO:0032268	regulation of cellular protein metabolic process	25	1683	0.000415
GO:0001819	positive regulation of cytokine production	10	280	0.0004326
GO:0006959	humoral immune response	9	226	0.0005615
GO:0051251	positive regulation of lymphocyte activation	8	170	0.000568
GO:0006820	anion transport	27	1963	0.0006176
GO:0048519	negative regulation of biological process	42	4017	0.0006248
GO:0010628	positive regulation of gene expression	24	1614	0.0006515
GO:0012501	programmed cell death	21	1283	0.0006755
GO:0043367	CD4-positive, alpha-beta T cell differentiation	5	47	0.0006869

GO:0031323	regulation of cellular metabolic process	47	4820	0.0007156
GO:0009615	response to virus	9	235	0.0007253
GO:2000026	regulation of multicellular organismal development	16	792	0.0007951
GO:0006897	endocytosis	11	376	0.0008498
GO:0033036	macromolecule localization	29	2254	0.0008498
GO:0009617	response to bacterium	10	310	0.0009622
GO:0031347	regulation of defense response	12	459	0.0009784
GO:0002520	immune system development	10	311	0.0009784
GO:0019058	viral life cycle	9	249	0.001099
GO:0070663	regulation of leukocyte proliferation	7	138	0.0012539
GO:0050792	regulation of viral process	8	193	0.0012813
GO:0031349	positive regulation of defense response	9	255	0.0012845
GO:0009611	response to wounding	11	395	0.0012845
GO:1903900	regulation of viral life cycle	7	139	0.0012845
GO:0071705	nitrogen compound transport	23	1575	0.0012953
GO:0080090	regulation of primary metabolic process	45	4616	0.0013153
GO:0051171	regulation of nitrogen compound metabolic process	44	4468	0.0013664
GO:1903037	regulation of leukocyte cell-cell adhesion	8	196	0.0013664
GO:1903039	positive regulation of leukocyte cell-cell adhesion	7	141	0.0013664
GO:0070727	cellular macromolecule localization	23	1585	0.0013899
GO:0046632	alpha-beta T cell differentiation	5	56	0.001412
GO:0071357	cellular response to type I interferon	5	56	0.001412
GO:0045619	regulation of lymphocyte differentiation	6	95	0.0014261
GO:0051240	positive regulation of multicellular organismal process	16	845	0.0015479
GO:0043903	regulation of biological process involved in symbiotic interaction	8	202	0.0016257
GO:0008104	protein localization	26	1975	0.0017709
GO:0034340	response to type I interferon	5	61	0.0020841
GO:0010605	negative regulation of macromolecule metabolic process	27	2126	0.0021864
GO:0051607	defense response to virus	7	154	0.002243
GO:0140546	defense response to symbiont	7	154	0.002243
GO:0046634	regulation of alpha-beta T cell activation	5	63	0.0023815
GO:0051239	regulation of multicellular organismal process	24	1767	0.0024019
GO:0007249	I-kappaB kinase/NF-kappaB signaling	8	216	0.0024903
GO:0000302	response to reactive oxygen species	6	106	0.0024903

GO:0032649	regulation of interferon-gamma production	5	64	0.002511
GO:0019079	viral genome replication	6	107	0.0025949
GO:0070488	neutrophil aggregation	2	2	0.0026033
GO:0032103	positive regulation of response to external stimulus	10	357	0.002633
GO:0043085	positive regulation of catalytic activity	16	889	0.002633
GO:0051235	maintenance of location	8	221	0.0028309
GO:0051173	positive regulation of nitrogen compound metabolic process	27	2166	0.0028369
GO:0080134	regulation of response to stress	17	1008	0.0032206
GO:0006413	translational initiation	7	165	0.0032347
GO:0032101	regulation of response to external stimulus	14	711	0.0032527
GO:0042886	amide transport	20	1338	0.0033001
GO:0032609	interferon-gamma production	5	69	0.0033846
GO:0051241	negative regulation of multicellular organismal process	13	622	0.0033846
GO:0034613	cellular protein localization	22	1576	0.0034718
GO:0022409	positive regulation of cell-cell adhesion	7	168	0.0035095
GO:0031325	positive regulation of cellular metabolic process	28	2334	0.0036736
GO:1901701	cellular response to oxygen-containing compound	13	634	0.0040296
GO:0044093	positive regulation of molecular function	18	1139	0.0040708
GO:0030099	myeloid cell differentiation	8	235	0.0040708
GO:0070661	leukocyte proliferation	7	175	0.0044267
GO:0071385	cellular response to glucocorticoid stimulus	3	14	0.0045275
GO:0060337	type I interferon signaling pathway	5	76	0.0051322
GO:0034341	response to interferon-gamma	6	124	0.0052871
GO:0010629	negative regulation of gene expression	22	1628	0.0053819
GO:0050670	regulation of lymphocyte proliferation	6	125	0.0054154
GO:0050870	positive regulation of T cell activation	6	125	0.0054154
GO:0070527	platelet aggregation	4	40	0.0054154
GO:0015031	protein transport	19	1281	0.0054502
GO:0032944	regulation of mononuclear cell proliferation	6	126	0.0056032
GO:0010942	positive regulation of cell death	10	398	0.0057221
GO:1902107	positive regulation of leukocyte differentiation	5	80	0.0062331
GO:1903708	positive regulation of hemopoiesis	5	80	0.0062331
GO:0007155	cell adhesion	16	968	0.0062726
GO:0060548	negative regulation of cell death	12	576	0.0063424
GO:0071384	cellular response to corticosteroid stimulus	3	16	0.0064322
GO:0045580	regulation of T cell differentiation	5	81	0.0064842

GO:0001818	negative regulation of cytokine production	7	189	0.0065895
GO:0022610	biological adhesion	16	975	0.0066611
GO:2000514	regulation of CD4-positive, alpha-beta T cell activation	4	43	0.0067619
GO:0050778	positive regulation of immune response	11	493	0.0067915
GO:0002684	positive regulation of immune system process	13	676	0.0068365
GO:0048523	negative regulation of cellular process	36	3564	0.0068893
GO:0045785	positive regulation of cell adhesion	8	258	0.006932
GO:0045069	regulation of viral genome replication	5	83	0.0069587
GO:0032496	response to lipopolysaccharide	6	133	0.0069587
GO:0010952	positive regulation of peptidase activity	6	133	0.0069587
GO:0015833	peptide transport	19	1320	0.0074156
GO:0050790	regulation of catalytic activity	20	1439	0.007566
GO:0050863	regulation of T cell activation	7	195	0.007566
GO:0043066	negative regulation of apoptotic process	11	504	0.0078748
GO:0031324	negative regulation of cellular metabolic	23	1815	0.008304
GO:0045582	positive regulation of T cell differentiation	4	46	0.0083052
GO:0019886	antigen processing and presentation of exogenous peptide antigen via MHC class II	5	87	0.008406
GO:0043372	positive regulation of CD4-positive, alpha-beta T cell differentiation	3	18	0.0085007
GO:0042542	response to hydrogen peroxide	4	47	0.0089172
GO:0022407	regulation of cell-cell adhesion	8	270	0.008938
GO:0065008	regulation of biological quality	31	2895	0.0089967
GO:0030334	regulation of cell migration	12	607	0.0092863
GO:0051651	maintenance of location in cell	6	142	0.0094012
GO:0043069	negative regulation of programmed cell death	11	517	0.0094153
GO:0002495	antigen processing and presentation of peptide antigen via MHC class II	5	90	0.0094807
GO:0002504	antigen processing and presentation of peptide or polysaccharide antigen via MHC class II	5	90	0.0094807
GO:0009056	catabolic process	25	2094	0.0095364
GO:0097190	apoptotic signaling pathway	9	352	0.0099626
GO:0002237	response to molecule of bacterial origin	6	146	0.010484
GO:0006935	chemotaxis	10	438	0.010484
GO:0042330	taxis	10	438	0.010484
GO:0045184	establishment of protein localization	19	1364	0.010484
GO:0044248	cellular catabolic process	23	1851	0.010484
GO:0050832	defense response to fungus	3	20	0.0109415
GO:0045589	regulation of regulatory T cell differentiation	3	20	0.0109415

GO:0031394	positive regulation of prostaglandin biosynthetic process	2	4	0.0110475
GO:0002819	regulation of adaptive immune response	5	94	0.0110569
GO:0048525	negative regulation of viral process	5	94	0.0110569
GO:0051345	positive regulation of hydrolase activity	10	450	0.0127183
GO:0045621	positive regulation of lymphocyte differentiation	4	53	0.012986
GO:0032602	chemokine production	4	53	0.012986
GO:0006886	intracellular protein transport	15	944	0.0135357
GO:0043170	macromolecule metabolic process	59	7578	0.0136492
GO:0045066	regulatory T cell differentiation	3	22	0.0141283
GO:0030155	regulation of cell adhesion	10	458	0.0142971
GO:0034109	homotypic cell-cell adhesion	4	55	0.0146957
GO:0002695	negative regulation of leukocyte activation	5	101	0.0149152
GO:0046651	lymphocyte proliferation	6	158	0.0151223
GO:0051172	negative regulation of nitrogen compound metabolic process	21	1659	0.0154577
GO:0032479	regulation of type I interferon production	5	102	0.0154577
GO:0032943	mononuclear cell proliferation	6	159	0.0154577
GO:0051336	regulation of hydrolase activity	13	749	0.0154577
GO:0045622	regulation of T-helper cell differentiation	3	23	0.0155542
GO:2000516	positive regulation of CD4-positive, alpha-beta T cell activation	3	23	0.0155542
GO:0032606	type I interferon production	5	103	0.0158348
GO:0032879	regulation of localization	23	1918	0.015983
GO:0071376	cellular response to corticotropin-releasing hormone stimulus	2	5	0.0165517
GO:0031340	positive regulation of vesicle fusion	2	5	0.0165517
GO:0043435	response to corticotropin-releasing hormone	2	5	0.0165517
GO:2001280	positive regulation of unsaturated fatty acid biosynthetic process	2	5	0.0165517
GO:0097012	response to granulocyte macrophage colony-stimulating factor	2	5	0.0165517
GO:0097011	cellular response to granulocyte macrophage colony-stimulating factor stimulus	2	5	0.0165517
GO:0050793	regulation of developmental process	20	1553	0.0170193
GO:0001776	leukocyte homeostasis	3	24	0.0170193
GO:0009620	response to fungus	3	24	0.0170193
GO:2000145	regulation of cell motility	12	662	0.017279
GO:0034121	regulation of toll-like receptor signaling	4	59	0.0177834

GO:0007275	multicellular organism development	31	3037	0.0182602
GO:0032677	regulation of interleukin-8 production	4	60	0.0187644
GO:0046718	viral entry into host cell	4	60	0.0187644
GO:0051384	response to glucocorticoid	3	25	0.0187974
GO:0032501	multicellular organismal process	43	4884	0.0189143
GO:0009719	response to endogenous stimulus	14	882	0.020149
GO:0046638	positive regulation of alpha-beta T cell differentiation	3	26	0.0209343
GO:0051247	positive regulation of protein metabolic process	16	1107	0.0209724
GO:0032637	interleukin-8 production	4	63	0.0221435
GO:0040012	regulation of locomotion	12	686	0.0228912
GO:0045647	negative regulation of erythrocyte	2	6	0.0230064
GO:0031392	regulation of prostaglandin biosynthetic process	2	6	0.0230064
GO:0033365	protein localization to organelle	13	790	0.0230064
GO:0050866	negative regulation of cell activation	5	114	0.0230064
GO:0002573	myeloid leukocyte differentiation	5	114	0.0230064
GO:0051090	regulation of DNA-binding transcription factor activity	8	324	0.0237904
GO:0032870	cellular response to hormone stimulus	8	324	0.0237904
GO:0006446	regulation of translational initiation	4	65	0.0241028
GO:0042742	defense response to bacterium	6	177	0.0241028
GO:0042981	regulation of apoptotic process	14	904	0.02456
GO:0071216	cellular response to biotic stimulus	5	117	0.0252495
GO:0030154	cell differentiation	27	2536	0.0252495
GO:0009725	response to hormone	9	413	0.0253731
GO:0002683	negative regulation of immune system process	7	250	0.0253731
GO:0060333	interferon-gamma-mediated signaling pathway	4	67	0.0264952
GO:0031960	response to corticosteroid	3	29	0.0272037
GO:0019730	antimicrobial humoral response	5	120	0.0277982
GO:0052547	regulation of peptidase activity	7	255	0.028084
GO:0002768	immune response-regulating cell surface receptor signaling pathway	8	335	0.0284566
GO:0019538	protein metabolic process	38	4188	0.028574
GO:0015671	oxygen transport	2	7	0.0292289
GO:0036490	regulation of translation in response to endoplasmic reticulum stress	2	7	0.0292289
GO:0043370	regulation of CD4-positive, alpha-beta T cell differentiation	3	30	0.0292289
GO:0018119	peptidyl-cysteine S-nitrosylation	2	7	0.0292289
GO:0032119	sequestering of zinc ion	2	7	0.0292289

GO:0030850	prostate gland development	2	7	0.0292289
GO:0035740	CD8-positive, alpha-beta T cell proliferation	2	7	0.0292289
GO:2001279	regulation of unsaturated fatty acid biosynthetic process	2	7	0.0292289
GO:0002755	MyD88-dependent toll-like receptor signaling pathway	3	30	0.0292289
GO:0002764	immune response-regulating signaling pathway	8	337	0.0292289
GO:0017014	protein nitrosylation	2	7	0.0292289
GO:2000564	regulation of CD8-positive, alpha-beta T cell proliferation	2	7	0.0292289
GO:0032056	positive regulation of translation in response to stress	2	7	0.0292289
GO:0043067	regulation of programmed cell death	14	928	0.0294285
GO:0044409	entry into host	4	71	0.0309266
GO:0002697	regulation of immune effector process	7	262	0.0310902
GO:0051270	regulation of cellular component movement	12	722	0.0318507
GO:0050671	positive regulation of lymphocyte proliferation	4	72	0.032098
GO:0050864	regulation of B cell activation	4	72	0.032098
GO:0048872	homeostasis of number of cells	4	72	0.032098
GO:0002699	positive regulation of immune effector process	5	127	0.0330719
GO:0045444	fat cell differentiation	5	127	0.0330719
GO:0010950	positive regulation of endopeptidase activity	5	127	0.0330719
GO:0032946	positive regulation of mononuclear cell proliferation	4	73	0.0333975
GO:0033993	response to lipid	8	348	0.0334749
GO:0042060	wound healing	8	348	0.0334749
GO:0019882	antigen processing and presentation	6	193	0.0335816
GO:0043112	receptor metabolic process	5	128	0.0337444
GO:0048856	anatomical structure development	33	3478	0.0347132
GO:0002460	adaptive immune response based on somatic recombination of immune receptors built from immunoglobulin superfamily domains	5	129	0.0347397
GO:0048869	cellular developmental process	27	2613	0.0359529
GO:1902563	regulation of neutrophil activation	2	8	0.0366132
GO:0006909	phagocytosis	6	197	0.0366951
GO:0045185	maintenance of protein location	4	76	0.0377045
GO:0043269	regulation of ion transport	13	848	0.0378503
GO:0032663	regulation of interleukin-2 production	3	34	0.0382437
GO:0046596	regulation of viral entry into host cell	3	34	0.0382437
GO:0034142	toll-like receptor 4 signaling pathway	3	34	0.0382437

GO:0031328	positive regulation of cellular biosynthetic process	17	1312	0.0386886
GO:0007229	integrin-mediated signaling pathway	4	77	0.0389039
GO:0034645	cellular macromolecule biosynthetic process	36	3967	0.0395029
GO:1901216	positive regulation of neuron death	3	35	0.0411732
GO:0044267	cellular protein metabolic process	35	3831	0.0430054
GO:0001525	angiogenesis	7	282	0.0439285
GO:0070665	positive regulation of leukocyte proliferation	4	80	0.0442181
GO:0032623	interleukin-2 production	3	36	0.0442181
GO:0045595	regulation of cell differentiation	14	978	0.0444339
GO:0009891	positive regulation of biosynthetic process	17	1332	0.044496
GO:0002468	dendritic cell antigen processing and presentation	2	9	0.044496
GO:0034695	response to prostaglandin E	2	9	0.044496
GO:0002822	regulation of adaptive immune response based on somatic recombination of immune receptors built from immunoglobulin superfamily domains	4	81	0.0455484
GO:0046637	regulation of alpha-beta T cell differentiation	3	37	0.0469766
GO:0046635	positive regulation of alpha-beta T cell activation	3	37	0.0469766
GO:0044070	regulation of anion transport	10	559	0.0471908
GO:0062207	regulation of pattern recognition receptor signaling pathway	4	83	0.0493199
GO:0009059	macromolecule biosynthetic process	36	4022	0.0495811

File S1 (b) TargetMine: Gene Ontology (Celleular Component)

Identifier	Name	Foreground	Background	p-value
GO:1903561	extracellular vesicle	34	1867	3.17E-08
GO:0043230	extracellular organelle	34	1868	3.17E-08
GO:0070062	extracellular exosome	34	1848	3.17E-08
GO:0031982	vesicle	44	3110	3.33E-08
GO:0030141	secretory granule	20	649	7.95E-08
GO:0005615	extracellular space	40	2702	7.95E-08
GO:0005576	extracellular region	43	3192	1.92E-07
GO:0031410	cytoplasmic vesicle	30	1706	5.50E-07
GO:0097708	intracellular vesicle	30	1707	5.50E-07
GO:0099503	secretory vesicle	20	763	6.69E-07
GO:0098576	lumenal side of membrane	5	24	0.0000259
GO:0035976	transcription factor AP-1 complex	3	3	0.0000259
GO:0060205	cytoplasmic vesicle lumen	11	267	0.0000278
GO:0034774	secretory granule lumen	11	265	0.0000278
GO:0031983	vesicle lumen	11	268	0.0000278
GO:0072562	blood microparticle	7	97	0.0001129
GO:0005925	focal adhesion	11	320	0.0001421
GO:0030055	cell-substrate junction	11	325	0.0001561
GO:0030139	endocytic vesicle	9	220	0.0003048
GO:0098553	lumenal side of endoplasmic reticulum membrane	4	20	0.0003279
GO:0071556	integral component of lumenal side of endoplasmic reticulum membrane	4	20	0.0003279
GO:0098552	side of membrane	11	377	0.0005316
GO:0042613	MHC class II protein complex	3	9	0.0010244
GO:0070161	anchoring junction	13	585	0.0012127
GO:0005775	vacuolar lumen	7	148	0.0012127
GO:0030669	clathrin-coated endocytic vesicle membrane	4	29	0.0012505
GO:0062023	collagen-containing extracellular matrix	9	282	0.0014701
GO:1904813	ficolin-1-rich granule lumen	6	104	0.0014701
GO:0030659	cytoplasmic vesicle membrane	12	516	0.0014701
GO:0042611	MHC protein complex	3	11	0.0014811
GO:0101002	ficolin-1-rich granule	7	158	0.0014811
GO:0098852	lytic vacuole membrane	9	290	0.0016042
GO:0005765	lysosomal membrane	9	290	0.0016042
GO:0009986	cell surface	12	531	0.0016042
GO:0012506	vesicle membrane	12	530	0.0016042

GO:0012505	endomembrane system	36	3534	0.0022903
GO:0005774	vacuolar membrane	9	320	0.0030843
GO:0045334	clathrin-coated endocytic vesicle	4	40	0.0031015
GO:0005770	late endosome	7	184	0.0031015
GO:1904724	tertiary granule lumen	4	43	0.0039837
GO:0071682	endocytic vesicle lumen	3	17	0.0045065
GO:0030665	clathrin-coated vesicle membrane	5	86	0.0045419
GO:0012507	ER to Golgi transport vesicle membrane	4	45	0.0045419
GO:0043233	organelle lumen	43	4850	0.0062655
GO:0005764	lysosome	11	537	0.0062655
GO:0031974	membrane-enclosed lumen	43	4850	0.0062655
GO:0000323	lytic vacuole	11	537	0.0062655
GO:0022626	cytosolic ribosome	5	95	0.0065133
GO:0005829	cytosol	41	4580	0.0077497
GO:0030666	endocytic vesicle membrane	5	108	0.0113782
GO:0005886	plasma membrane	39	4346	0.0115195
GO:0031012	extracellular matrix	9	399	0.0115195
GO:0005773	vacuole	11	596	0.0135964
GO:0031838	haptoglobin-hemoglobin complex	2	6	0.0146883
GO:0071944	cell periphery	39	4424	0.0160422
GO:0031091	platelet alpha granule	4	69	0.0177281
GO:0005737	cytoplasm	67	9567	0.0177281
GO:0031232	extrinsic component of external side of plasma membrane	2	7	0.0189247
GO:0030134	COPII-coated ER to Golgi transport vesicle	4	71	0.0189247
GO:0030054	cell junction	16	1200	0.0217806
GO:0031090	organelle membrane	26	2526	0.0221029
GO:0043227	membrane-bounded organelle	71	10499	0.0222498
GO:0016020	membrane	53	6930	0.0222498
GO:0030662	coated vesicle membrane	5	134	0.0227576
GO:0005833	hemoglobin complex	2	8	0.0227576
GO:0009897	external side of plasma membrane	6	201	0.0227576
GO:0043202	lysosomal lumen	4	77	0.0227576
GO:0030176	integral component of endoplasmic reticulum membrane	5	135	0.0231861
GO:0098588	bounding membrane of organelle	18	1474	0.0237391
GO:0030136	clathrin-coated vesicle	5	139	0.025679

GO:0070820	tertiary granule	5	141	0.026989
GO:0031227	intrinsic component of endoplasmic reticulum membrane	5	142	0.027468
GO:0022627	cytosolic small ribosomal subunit	3	38	0.0288973
GO:0005911	cell-cell junction	7	297	0.0296924
GO:0030660	Golgi-associated vesicle membrane	4	87	0.0319142
GO:0005802	trans-Golgi network	6	222	0.0326822
GO:0032588	trans-Golgi network membrane	4	89	0.0338165
GO:0010008	endosome membrane	7	307	0.0340748
GO:0005634	nucleus	47	6038	0.0367039

File S1 (c) TargetMine: Reactome

Identifier	Name	Foreground	Background	p-value
R-HSA-168256	Immune System	60	1970	4.69E-15
R-HSA-168249	Innate Immune System	41	1044	2.30E-12
R-HSA-6798695	Neutrophil degranulation	27	479	6.13E-11
R-HSA-1280215	Cytokine Signaling in Immune system	32	699	6.13E-11
R-HSA-913531	Interferon Signaling	16	194	3.62E-08
R-HSA-6785807	Interleukin-4 and Interleukin-13 signaling	11	108	0.0000037
R-HSA-202427	Phosphorylation of CD3 and TCR zeta chains	6	21	0.0000146
R-HSA-114608	Platelet degranulation	11	128	0.0000166
R-HSA-76005	Response to elevated platelet cytosolic Ca2+	11	133	0.000022
R-HSA-2173782	Binding and Uptake of Ligands by Scavenger Receptors	7	41	0.0000342
R-HSA-877300	Interferon gamma signaling	9	89	0.0000518
R-HSA-168898	Toll-like Receptor Cascades	11	151	0.0000605
R-HSA-909733	Interferon alpha/beta signaling	8	68	0.0000651
R-HSA-449147	Signaling by Interleukins	18	460	0.0001045
R-HSA-202430	Translocation of ZAP-70 to Immunological synapse	5	18	0.0001154
R-HSA-389948	PD-1 signaling	5	22	0.0003221
R-HSA-1236977	Endosomal/Vacuolar pathway	4	11	0.0004285
R-HSA-109582	Hemostasis	19	598	0.000818
R-HSA-2168880	Scavenging of heme from plasma	4	13	0.000818
R-HSA-76002	Platelet activation, signaling and aggregation	12	259	0.0012318
R-HSA-202433	Generation of second messenger molecules	5	33	0.0020267
R-HSA-202403	TCR signaling	8	119	0.0026826
R-HSA-202424	Downstream TCR signaling	7	97	0.0051552
R-HSA-9613829	Chaperone Mediated Autophagy	4	21	0.0051552
R-HSA-983170	Antigen Presentation: Folding, assembly and peptide loading of class I MHC	4	24	0.0083885
R-HSA-2132295	MHC class II antigen presentation	7	106	0.0083885
R-HSA-1169410	Antiviral mechanism by IFN-stimulated genes	6	77	0.0105493
R-HSA-1679131	Trafficking and processing of endosomal TLR	3	13	0.0227408
R-HSA-1280218	Adaptive Immune System	18	723	0.0227408
R-HSA-1236975	Antigen processing-Cross presentation	6	98	0.0355974
R-HSA-388841	Costimulation by the CD28 family	5	65	0.0370936
R-HSA-5686938	Regulation of TLR by endogenous ligand	3	16	0.0382003
R-HSA-9031628	NGF-stimulated transcription	4	39	0.0443713
R-HSA-1169408	ISG15 antiviral mechanism	5	69	0.0445877
